# Improving quality of life and self-care for patients on hemodialysis using cognitive behavioral strategies: A randomized controlled pilot trial

**DOI:** 10.1371/journal.pone.0285156

**Published:** 2023-05-04

**Authors:** Shayan Shirazian, Arlene M. Smaldone, Alan M. Jacobson, Melissa J. Fazzari, Katie Weinger

**Affiliations:** 1 Division of Nephrology, Department of Medicine, Columbia University Medical Center, New York, New York, United States of America; 2 Department of Nursing, Columbia University School of Nursing, New York, New York, United States of America; 3 Research Institute, NYU Long Island School of Medicine, Mineola, New York, United States of America; 4 Department of Epidemiology & Population Health, Albert Einstein College of Medicine, Bronx, New York, United States of America; 5 Joslin Diabetes Center, Harvard Medical School, Boston, Massachusetts, United States of America; Northwell Health Feinstein Institutes for Medical Research, UNITED STATES

## Abstract

**Introduction:**

Behavioral-education interventions have the potential to improve quality of life and self-care for patients on hemodialysis (HD) but have not been incorporated into routine clinical practice. The purpose of this pilot study was to determine the feasibility of delivering a simple behavioral-education intervention using cognitive behavioral strategies in patients receiving HD with poor quality of life.

**Methods:**

In this mixed methods study, HD patients were randomly assigned to the study intervention (8 behavioral-education sessions delivered over 12 weeks) or a control group of dialysis education alone. Kidney disease quality of life (KDQOL)-36 scores, depressive symptoms and self-care behaviors were measured at weeks 0, 8, and 16. Following study completion, participants, social workers, and physicians provided their perspectives about the intervention via qualitative interviews.

**Findings:**

Forty-five participants were randomized. Due, in part, to social worker attrition from the intervention arm, 34 participants (76%) completed at least 1 study session and were included in the analysis. The intervention led to modest, but non-significant, increase in KDQOL-physical component summary scores (+3.1±1.2 points) from week 0 to week 16. There were small, non-significant decreases in interdialytic weight gain and pre-dialysis phosphorus levels in the intervention group. Participants felt that chair-side delivery was practical and efficient, and that content related to the impact of dialysis on daily life was unique and important. Suggestions for adapting the intervention included narrowing its content and its delivery by additional providers that are not necessarily therapy trained.

**Discussion:**

In this pilot study, we were able to deliver a simple behavioral-education intervention to improve both quality of life and self-care. Participants had a positive impression of the intervention, but we did not find significant improvements in quality of life or self-care. We will now adapt our intervention by narrowing its content and by using other providers that are focused solely on delivering the intervention.

## Introduction

Quality of life (QOL) for patients on hemodialysis (HD) is significantly worse than the general population and is among the worst of any chronic illness [1]. Worse QOL scores are associated with a higher risk of hospitalizations and death [2].

Self-care, the active participation of patients in their treatment, is critical for the physical and psychosocial well-being of patients on HD and involves adherence to medications, dialysis, diet, activity, self-monitoring and physician follow-up [3]. Poor quality of life and self-care are closely linked. For patients on HD, it is likely that the association between adverse medical outcomes and poor QOL is, in part, mediated by poor self-care. QOL has been consistently associated with higher phosphorus and potassium levels, and higher interdialytic weight gain [4–6] which, in turn, have been associated with hospitalizations and higher mortality [7–9].

In patients on HD, cognitive behavioral therapy (CBT)-based interventions delivered by psychologists have successfully treated depression (a major component of QOL) and led to secondary improvements in self-care [10, 11]. Although promising, the majority of these studies enrolled select HD populations (i.e. only those with depression) and required HD centers to hire psychologists. Few studies of behavioral-education interventions, applicable to the majority of patients on HD, and focused on improvements in QOL and self-care have been performed [12, 13].

We developed a behavioral-education intervention to improve QOL and self-care in patients on HD. The intervention involves presenting a one-page dashboard that displays recent QOL results and self-care indicators to the HD treatment team and then developing an individualized treatment approach that combines self-care education with cognitive-behavioral (CB) strategies through 8 sessions delivered chair-side by a HD social worker over 12 weeks. The purpose of this pilot study was to determine the feasibility of its implementation and its impact on QOL and self-care.

## Materials and methods

The study was IRB approved by the institutional review board at Columbia. Written informed consent was obtained from all subjects and all procedures followed were in accordance with the Declaration of Helsinki.

### Study design

This study was designed as a 16-week randomized-controlled pilot trial in which 45 patients on HD with kidney disease quality of life (KDQOL) scores below 50 were randomized 1:1 to the intervention vs. control. Written informed consent was obtained from all subjects.

Randomization procedures were performed through REDCap ^®^. The study statistician provided a randomization schedule based on a total sample size of n = 48 with 1:1 allocation. Randomization was conducted using simple block randomization (block size = 4).

The study team, including the social workers that delivered the intervention, was aware of the randomization allocation but the patient’s treatment team and study subjects were not explicitly made aware. Subjects from both groups continued to receive medical care from their treating health care providers.

### Sample size

To examine the power to detect clinically relevant intervention-related changes in HrQOL outcomes, we simulated normally distributed pre and 16-week post intervention HrQOL scores with no changes in mean HrQOL in the control group and an absolute increase of at least 8 points in the MCS or PCS (from pre to post)in the intervention, a within-group standard deviation (SD) of 10.0 and positive correlation between HrQOL measurements from the same person such that Pearson’s rho = 0.70. Our hypothesized effect size was based on studies showing that a change of 8 points or more in PCS has been associated with a clinically significant decrease in hospitalizations and mortality [2, 14]. Behavioral interventions have shown similar improvements in HrQOL over 3–4 months [15, 16]. With n = 20 per treatment arm, we have 85% power at the 0.025 level of significance to detect clinically relevant changes in HrQOL. Given a presumed drop-out of 20%, we planned to randomize 48 participants. Due to difficulties in study recruitment, only 45 participants were ultimately randomized.

### Setting and study population

This study was conducted at two HD units in Manhattan, New York. Eligibility criteria included: 1) HD duration > 3 months; 2) Most recent 36-question kidney disease quality of life (KDQOL-36^™^) physical component summary (PCS) or mental component summary (MCS) score < 50; 3) KDQOL-36 burden of disease score ≤ 80; 4) Expected survival ≥ 6 months; 5) English-speaking.

Exclusion criteria included: 1) Bipolar or Psychotic disorder 2) Moderate or severe cognitive impairment as determined by the HD staff or documented in the electronic medical record 3) Severe vision or hearing impairment 4) Drug or alcohol dependence; 5) Active suicidal ideation or a history of suicide attempt.

### Intervention

The intervention has previously been described in detail [17]. In brief, the intervention had 3-steps. The first step was the creation of a one-page 3-section dashboard displaying recent QOL results, self-care indicators and proposed treatment to patients, family members and the HD treatment team during monthly case conferences.

The second step was an approach to improving self-care with education combined with CB strategies, adapted from a similar intervention in patients with diabetes [16]. Licensed clinical social workers employed in HD units implemented the eight, 20–30 minute sessions. Social workers underwent a 6-h training session in CB-strategies led by study investigator KW who had experience training educators in CB-strategies [16].

The intervention sessions were delivered one on one, chair-side. Family members were invited to attend. The curriculum included education relevant for self-care and incorporated CB strategies to overcome barriers to self-care including: 1) review of self-care logs, 2) goal setting, 3) creation of treatment plan, 4) problem-solving techniques, 5) reinforcing techniques, and 6) coping with end-stage kidney disease (ESKD). All sessions ended with individualized homework assignments. A log of completed homework assignments was kept. In order to improve fidelity, CB session slides were accompanied by a script and instructions on how and when to deliver worksheets and handouts during each session. Social workers were required to complete a checklist that ensured certain cognitive behavioral components were incorporated into each session including reviewing homework, addressing barriers to self-management, addressing negative emotions and cognitive distortions, and completing a behavioral shaping exercise. This checklist was reviewed by the PI to ensure completion. We had initially planned to tape sessions and review these tapes to ensure fidelity, however we could not do this due to a policy against audiotaping at one of hemodialysis centers.

The final step was monthly re-evaluation to determine if treatment variation was needed.

### Control

The control group received the current care for low QOL scores as dictated by HD unit policy. This included social work review of QOL scores and use of a template to report scores and suggestions back to patients. The control group also received educational sessions and materials without embedded CB-strategies. The control intervention was originally delivered by a social-worker, however, she left the institution and the control sessions were subsequently delivered by either a study social worker, dialysis tech or research assistant that was not involved in the delivery of the study intervention. They received the same amount of contact (eight, 20–30 minute sessions) with investigators as the intervention group.

### Outcomes

1. **Quality of life** measured using the KDQOL-36^™^ at weeks 0, 8 and 16 [18].
2. **Self-care** was measured by mean serum potassium (measured monthly), serum phosphorus (measured monthly) and interdialytic weight gain (IDWG) over the 3 months prior to and the 3 months during the intervention. Interdialytic weight gain was measured as the average weight gain between hemodialysis sessions (measured pre-dialysis). Thus, there were 2 values, one at week 0 and one at week 16, that were the average of approximately 39 readings.
3. **Depressive symptoms** measured using the Nine-Question Patient Health Questionnaire (PHQ-9) at weeks 0, 8 and 16 [19].

### Qualitative assessment

Following study completion, intervention participants, social workers and physicians with exposure to the intervention, but not part of the study team, were recruited to participate in a qualitative interview on the acceptability of the intervention and its potential for implementation to practice. Participant interviews were designed as in-person focus groups; however, due to subject concerns of COVID transmission from in-person gatherings, participants were given the option of participating in individual semi-structured interviews over the phone. All participants chose individual semi-structured interviews. Interviews used an established interview guide [17], and lasted between 20–60 minutes. Interviews were digitally audio-recorded, transcribed and reviewed to assure accuracy. All interviews were conducted by a qualitative researcher without exposure to the intervention or control.

### Feasibility assessment

The feasibility of performing a larger trial and of implementing this intervention was determined through the collection of data related to: 1) the study eligibility of screened patients, 2) reasons for study exclusion and drop-out, 3) detailed logs of completed homework by participants, and 4) detailed logs of CB-strategies used during intervention sessions. Additionally, qualitative interviews of social workers and physicians that either administered or were exposed to the study intervention were performed, as above, to gauge the feasibility of the intervention.

### Data analysis

Baseline characteristics were compared between the intervention and control groups using independent t-test, Welch’s independent t-test, and two-tailed Fisher’s exact test as appropriate. Normality was tested using the Skewness/Kurtosis test. All compared baseline variables had a normal distribution.

For outcome measures, only those that completed at least one intervention or control session were analyzed given high drop-out. For participants that were included in the final analysis, 7.0% of KDQOL, PHQ-9 or self-care data was missing. For participants that had at least one study follow-up, but then subsequently did not complete the study (n = 5), the pattern of missing data was considered monotone. For participants that completed all study procedures, data was determined to be missing at random. For these participants only 0.25% of data was missing. Missing KDQOL and PHQ-9 measures were imputed using the last observation carried forward (LOCF). Missing self-care outcomes were left blank. For each QOL outcome, average changes from pre to post, were summarized via means (±SD) and 95% confidence intervals. Mean absolute differences between outcome scores pre-post intervention (week 0 and week 16) were compared within groups using paired t-tests and between groups using independent t-test or with an appropriate non-parametric test. The effect of our intervention was also estimated using a linear model for each post-treatment KDQOL score, adjusting for pre-treatment score and treatment arm (ANCOVA) and using a mixed effects sensitivity model examining the linear trend of MCS and PCS scores over week 0,8 and 16 and a group by time interaction. LOCF was used for the ANCOVA model, while missing outcomes were left blank for a mixed effects model sensitivity analysis. A 2-tailed alpha level of 0.05 was used for all analyses. All statistical analyses were conducted using Stata SE, version 15.0 (StataCorp, College Station, TX).

For the semi-structured interviews, transcripts were coded independently by three team members for identification of initial themes and then checked for inter-rater agreement. Content analysis conducted by marking and categorizing key words and phrases to identify themes of relevance to study participants [20, 21]. Themes were reviewed, discussed, and resolved through consensus. Data analysis occurred concurrently with the interview process thereby utilizing constant comparative analysis that allowed for identification of links across themes [22]. The research team met to review transcript coding, achievement of data saturation, consensus regarding identification and definition of themes, and selection of illustrative excerpts from transcripts. Dependability of the data interpretation was supported by investigator triangulation, a process where more than one investigator analyzes data through the use of an interdisciplinary team consisting of a nurse researcher, and physician with backgrounds in HD and qualitative research and a research assistant [23, 24]. Dependability was established through development and use of a codebook to reflect code definitions and theme identification as well as maintenance of an audit trail of reflexive and operational memos.

## Results

### Flowchart of study inclusion

**Fig 1:**
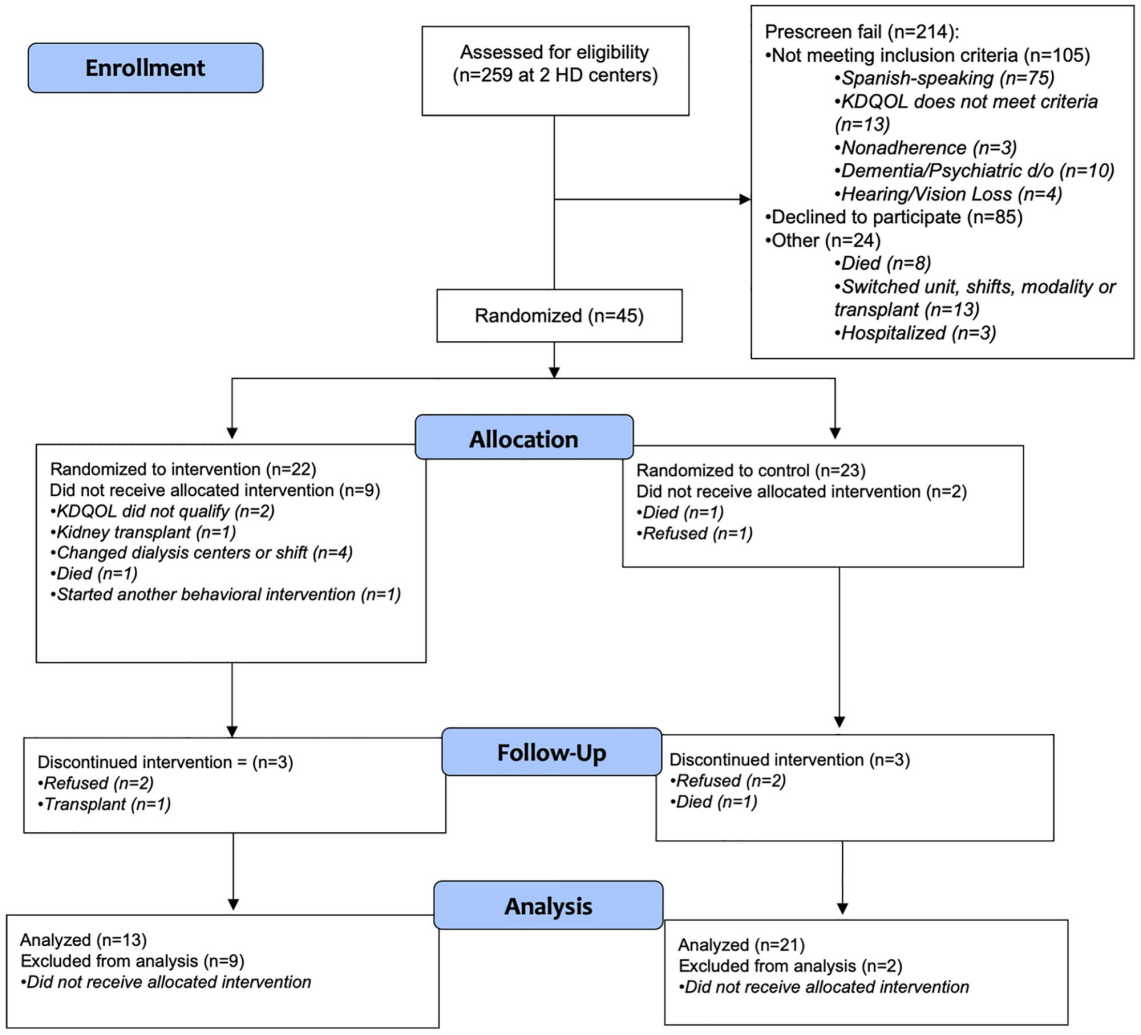
Flow chart of study inclusion.

The flowchart of study inclusion is shown in (Fig 1). Four social workers were trained to deliver the study intervention; however, two dropped out prior to performing any study sessions because of the additional work burden. This dropout delayed the time to session 1, and the average time to session 1 was 79 ± 52 days compared to 32 ± 30 days in the control. Twenty-two participants were randomized to the intervention group and 23 to the control. Nine participants dropped out prior to session 1 in the intervention while 2 dropped out in the control. Baseline characteristics of those randomized who did not complete session 1 were not significantly different from study participants. The most common intervention sessions were 2 sessions focused on coping with dialysis. Homework was completed 51% of the time (64% control and 26% intervention). No family members attended any sessions during the study period.

### Baseline characteristics

Baseline characteristics of the groups are shown in Table 1. There were no significant differences between the intervention and control group in baseline characteristics.

**Table 1:** Baseline characteristics of the study population.

Variable	Total (n = 34)	Intervention (n = 13)	Control (n = 21)	*p*-value
*Demographic/Socioeconomic Characteristics*				
Age (yr)	59.9±14.3	55.8±11.3	62.5 ± 15.5	0.18
Female	17 (50.0%)	7 (53.8%)	10 (47.6%)	1.00
Ethnicity Hispanic	7 (20.6%)	2 (15.4%)	5 (23.8%)	0.68
Racial/ethnic group				0.12
non-Hispanic White	9 (26.5%)	1 (7.7%)	8 (38.1%)	
non-Hispanic Black	23 (67.6%)	11 (84.6%)	12 (57.1%)	
American Indian/Alaska Native	2 (6.3%)	1 (7.6%)	1 (4.8%)	
Educational attainment				1.00
High School or lower	12 (35.3%)	4 (30.8%)	8 (38.1%)	
Associate	10 (29.4%)	4 (30.8%)	6 (28.6%)	
College or higher	12 (35.3%)	5 (38.5%)	7 (33.3%)	
Employment status				0.31
Employed	7 (20.5%)	4 (30.8%)	3 (14.3%)	
Not Employed	11 (32.3%)	5 (38.5%)	6 (28.6%)	
Retired	16 (47.1%)	4 (30.8%)	12 (57.1%)	
*Clinical Characteristics*				
Current Smoker	6 (17.6%)	1 (7.7%)	5 (23.8%)	0.37
Hypertension	26 (76.5%)	10 (76.9%)	16 (76.2%)	1.00
Diabetes	15 (44.1%)	7 (53.8%)	8 (38.1%)	0.48
CVD	13 (38.2%)	3 (23.1%)	10 (47.6%)	0.28
Total # of meds	8.6±(4.3)	7.5±2.6	9.3±5.0	0.20^a^
KDQOL-36 Subscales				
MCS	46.1±11.8	47.1±10.5	45.4±12.7	0.70
PCS	37.2±9.9	35.8±8.6	38.1±10.7	0.53
Burdens	36.4±20.9	40.5±22.1	34.0±20.2	0.19
Effects	58.8±21.1	61.3±17.4	57.2±24.4	0.29
Symptoms	78.3±(16.1)	79.6±12.0	77.4±18.5	0.35
PHQ-9	5.3±4.5	4.5±3.7	5.9±4.9	0.39
*Laboratory Characteristics*				
IDWG (lbs)	4.1±2.1	3.8±1.6	4.2±2.3	0.61
K (meq/l)	4.7±0.7	4.7±0.5	4.8±0.8	0.61
Phos (mg/dl)	5.7±1.5	6.3±1.1	5.3±1.6	0.07

Data are presented as n (%), mean ± standard deviation or median (interquartile range).

Note. yr, year; CVD, cardiovascular disease; #, number; KDQOL, kidney disease quality of life-36 question survey; MCS, mental component summary score; PCS, physical component summary score; PHQ-9, patient health questionnaire-9; IDWG, mean weight gain between hemodialysis sessions in the 3 preceding months; lbs, pounds; K, mean pre-HD serum potassium values in the 3 preceding months; Phos, mean pre-HD serum phosphorus values in the 3 preceding months; wk, week.

Groups with equal variance compared with independent t-test and when unequal variance, Welch’s independent t-test. Categorical variables were compared using two-tailed Fisher’s exact tests.

^a^, samples with unequal variance and Welch’s test performed.

### Quality of life (Fig 2, Tables 2 and 3)

**Fig 2:**
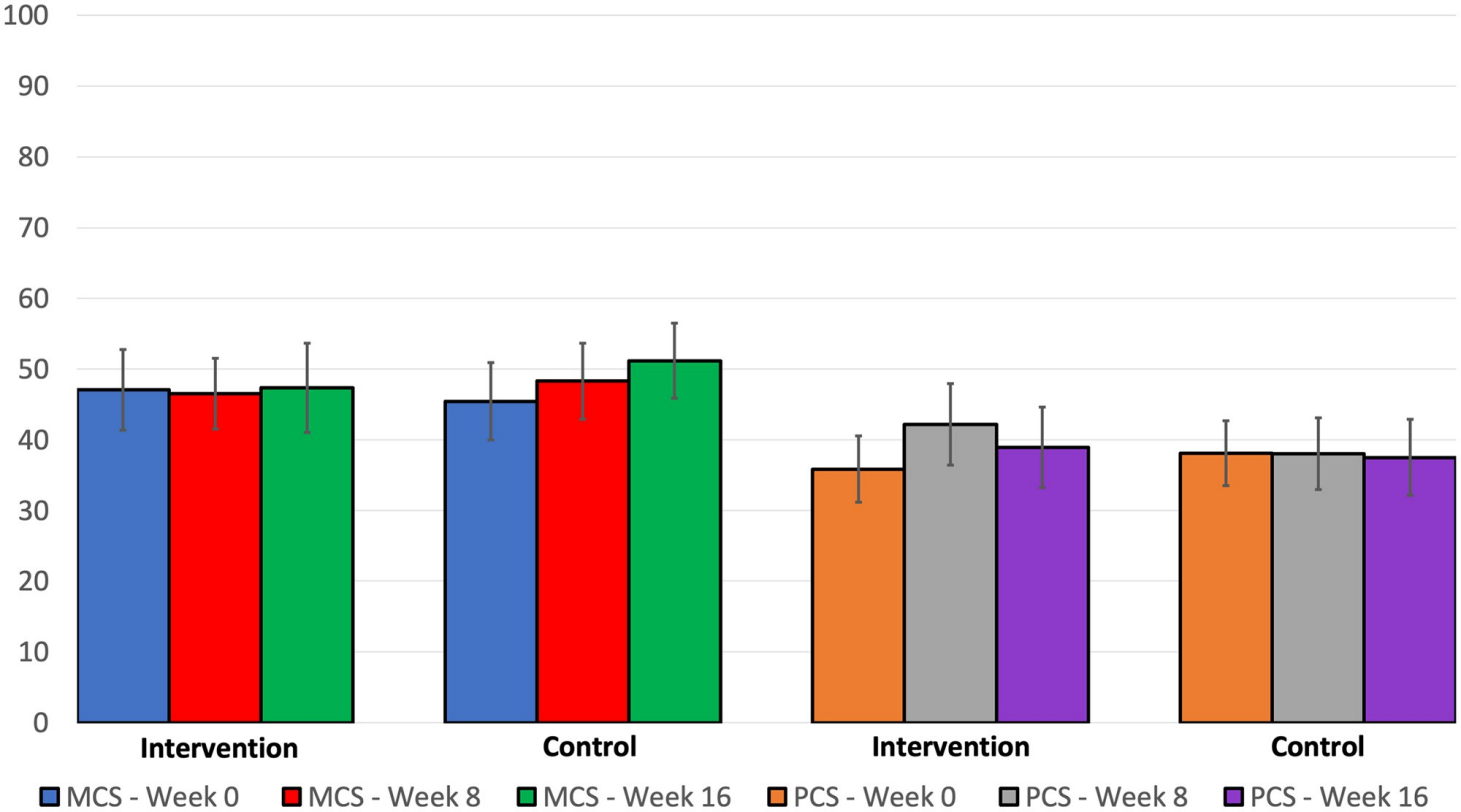
Change in quality of life over time. Note. MCS, mental component summary score; PCS, physical component summary score. Error bars represent 95% confidence intervals.

**Table 2:** Comparison of Week 0 and Week 16 outcomes in the intervention and control groups.

	Intervention (n = 13)			Control (n = 21)		
Outcome	Week 0	Week 16	Mean Difference (0–16 wk)	Week 0	Week 16	Mean Difference (0–16 wk)
*Quality of life surveys*						
KDQOL-36 Subscales						
MCS	47.1±10.5	47.4±11.6	0.3±10.9	45.4±12.7	55 (51–58)^b^	5.7±11.6
PCS	35.8±8.6	38.9±10.5	3.1±9.2	38.1±10.7	37.5±12.6	-0.60±9.4
Burdens	40.5±22.1	37.1±18.0	-3.4±24.1	34.0±20.2	41.4±21.8	7.4±18.0
Effects	61.3±17.4	62.0±11.9	0.7±18.5	57.2±24.4	63.0±24.8	5.8±17.4
Symptoms	79.6±12.0	79.9±11.0	0.3±8.8	77.4±18.5	80.1±15.6	2.7±9.9
PHQ-9	4.5±3.7	5.9±4.2	1.4±3.4	5.9±4.9	7.1±7.2	1.2±4.6
*Self-care markers*						
IDWG (lbs) ^a^	3.8±1.6	3.3±1.4	-0.9±1.7	4.2±2.3	4.0±3.1	-0.4±3.2
K (meq/l) ^a^	4.7±0.5	4.7±0.6	0.1±0.3	4.8±0.8	4.5±0.5	-0.2±0.7
Phos (mg/dl) ^a^	6.3±1.1	5.8±1.6	-0.5±1.3	5.3±1.6	5.4±2.0	0.03±1.8

Data are presented as mean ± standard deviation or median (interquartile range).

Note. KDQOL, kidney disease quality of life-36 question survey; MCS, mental component summary score; PCS, physical component summary score; PHQ-9, patient health questionnaire-9; IDWG, mean weight gain between hemodialysis sessions in the 3 preceding months; lb, pounds; K, mean pre-HD serum potassium values in the 3 preceding months; Phos, mean pre-HD serum phosphorus values in the 3 preceding months; wk, week.

Groups with equal variance compared with independent t-test and when unequal variance, Welch’s independent t-test.

^a^, values not imputed, 11 participants completed the intervention and 18 the control.

^b^, p<0.05 with-in group change using paired t-test.

**Table 3:** Adjusted group effect* for Week 16 PCS and MCS scores.

	Estimated group difference (95% CI)	p-value	Adjusted R^2^
PCS	3.15 (-2.96, 9.16)	0.34	0.38
MCS	-4.75 (-11.54, 2.04)	0.20	0.28

Note. PCS, physical component summary score; MCS, mental component summary score.

*Effect of intervention vs control, adjusted for pre-intervention score.

Week 0 and week 16 KDQOL subscale scores for the intervention and control groups are displayed in Table 2. MCS increased +0.3±10.9 points (95% CI: -5.6, 6.2) and PCS increased +3.1±1.2 points (95% CI: -1.9, 8.1) from week 0 to week 16 in the intervention arm. The mean week 0 to 16 difference in MCS scores, PCS scores, KDQOL-36 subscales, PHQ-9 scores and self-care markers were not statistically different between the intervention and control groups. The 95% CI for differences between the intervention and control groups in mean MCS scores was -2.69 to 13.63, and for PCS was -10.38 to 3.04. The difference in the arms for PCS corresponded to a small‐moderate mean effect size (Cohen’s d = 0.4). Within-group changes in outcomes were compared using paired t-tests. Only MCS scores increased significantly within the control group, otherwise there were no significant within-group changes in any outcomes. Group assignment was not related to week 16 MCS or PCS scores when controlling for pre-treatment scores (Table 3). A mixed effects model, without imputed missing values, examining a linear trend of MCS and PCS scores over week 0,8 and 16 and a group by time interaction was performed as a sensitivity analysis. The group by time parameter was not statistically significant indicating no effect of the intervention on MCS or PCS scores (Table 4). There were no significant differences in any outcome measures from Week 0 to Week 8 (data not shown).

**Table 4:** Estimated *Group x time* interaction effects for PCS and MCS score models*.

	Estimate (SE)	p-value
PCS	0.134 (0.22)	0.55
MCS	- 0.291 (0.23)	0.22

Note. PCS, physical component summary score; MCS, mental component summary score.

*Random intercept model for PCS or MCS, adjusted for the main effects of group and week, along with group x week interaction.

### Self-care markers and depressive symptoms (Table 2)

Mean week 0 to week 16 differences in self-care markers and depressive symptoms are shown in Table 2. There were no statistical differences between groups. In the intervention arm, there was a 0.9lb decrease in IDWG from week 0 to week 16 as compared to a 0.4lb decrease in the control. Phosphorus levels decreased by 0.5±1.3 meq/l in the intervention group, but this did not reach significance (p = 0.21), while increasing slightly in the control group (p = 0.94).

### Qualitative study

Ten participants completed all intervention sessions and were eligible for end-of-study qualitative interviews. One participant from each site consented to be interviewed. The most common reason for refusal was the ongoing COVID pandemic. Two social workers who delivered the intervention and three physicians were interviewed. Interviews were conducted either at the HD center or via video chat during the pandemic. Three themes were identified: 1) Chairside delivery: Convenient but private, 2) Engagement, and 3) Changing habits.

**1) Chairside delivery: Convenient but private**. Although physicians and social workers described chairside intervention delivery as not ideal because of the background noise level and open nature of the HD unit, all felt it was the most feasible approach for this setting because patients on HD "look to just be able to come dialyze and go home" (social worker site 2—SWS2), and "you have somewhat of a captive audience because they’re attached to a machine (SWS2).”

A physician at site 2 (PS2) described the pros and cons of chairside intervention delivery: "Patients feel enough time is being spent ’sucked away at dialysis’… they’re doing dialysis and getting this … two for the price of one at the same time and that’s actually a positive thing … it sort of decreases, you know, the privacy issue (PS2)."

Social workers described what they did to assure privacy for their conversations with study subjects. "I physically put myself between the two patients so they can no longer make eye contact and then the conversation is more between us… you know, low voices, making eye contact, sitting on a stool… you know, not standing in front of the patient to do it, so that you … have a little more of an intimate conversation. But, yeah, it’s not easy to do (SWS1)." Patients expressed that conducting the intervention sessions chairside “…made the time go by faster…” (subject No. 1-Female at site 1—S1F S1) and “…was actually something to do while I was sitting there (subject No. 2-Male at site 1- S2M S1).”

**2) Engagement**. Participants described engagement in various ways: a patient’s motivation to improve their health, optimizing the encounter during the dialysis session, and extending engagement to home. Sessions were described by one physician as "fill(ing) a gap for… for aspects of the patients’ personal life and how it’s impacted by their kidney disease and being on dialysis that we don’t specifically address (PS1)." Both patient participants described their participation as "something to do while sitting there (subject No. 3-Male at site 2 –S3M S2).” Social workers attempted to find the right time to deliver the intervention within the confines of a HD treatment: "Quite often people are very sleepy and so you have to try and catch them … before they’ve fallen asleep and, also, sometimes they’re quite tired or their cramping at the end of treatment or they’re nauseous or other things. So… there’s like kind of a sweet spot when you can meet (SWS1).” One patient found the homework very useful. "I got the homework every time so that was good, too. So I’m not just thinking about it while I’m here. You go home and think about each section (S3M S2).”

The perception of level of patient engagement varied. The patient "wanted to meet after sessions, not during sessions, because she sleeps the entire sessions and it’s really impossible to meet her chairside. And she’s like, no, no, I wanna keep doing it. But you could just tell she wasn’t really engaged (SWS1).”

"(He) was very motivated, would share a lot of goals, would do the homework, shared a lot about some personal aspects of his diagnosis, some of the challenges, some of the things that have been able to improve (SWS2).”

**3) Changing habits**. Dietary habits were frequently discussed, particularly related to phosphorus intake. Habits were viewed as hard to change and possibly taking longer to achieve. "It takes time, one, for the person giving the therapy to break down barriers and connect, and then to make a difference… I wonder if you need a longer period of time (PS2)."

Patients varied regarding how they perceived their motivation and/or willingness to change behaviors. “Diet is constantly a problem and I… I fight that phosphorous all the time. And, uh, I cook for myself and I just listen to anything… I’m sure I took things home with me from this program" yet didn’t make "not too much change… I’m not really big on changes (S1F S1)." This contrasted to the behavior change another patient, "if I’m just sittin’ at home I’m eating but if I’m doing… keeping occupied … then not so much drinking or eating the wrong things. So that’s how I avoid it… Like going out, just hanging out with friends, just doing chores and I recently did start back working like a day or two (S3M S2)."

#### Suggested changes

While overall perceptions of the intervention and its utility were positive, a social worker at one study site as well as physicians at both sites made suggestions to improve feasibility. For example, intervention content was felt to be too broad with “a lot more material in it than would ever be able to be covered in the time period I was allotted to meet with patients (SWS1).” Notably, the social worker wished that “we could have had less of the education piece and more of the CB intervention piece. It’s just hard to do a twenty to thirty-minute session and do all this education and then sort of squeak in a little bit of therapy at the end. I just felt like it was lopsided in that way (SWS1).” Physicians acknowledged that they had very limited knowledge of intervention content but did have suggestions regarding alternatives to social workers for intervention delivery. In the words of one physician “… the biggest prerequisite for giving this therapy for the patient is you have to be a person who legitimately cares about the other person. I don’t think you need a degree (PS2).” Another physician reiterated this idea by saying “I think it would not have to include the teammates that are here already doing a different job, and that’s something that usually happens, so if we want it (the intervention) to be successful, it has to be driven by someone who is specifically doing this particular job (PS1).”

## Discussion

In this pilot trial we tested the feasibility of implementing a behavioral-education intervention for patients on HD, and the effect of our intervention on QOL and self-care. We were only able to complete the study in 29 participants despite assessing 259 patients for eligibility. For participants that did complete the study, there was a positive impression of the intervention. Participants liked that it was delivered during the HD session and its focus on quality of life. Our intervention did not lead to any significant change in KDQOL-36 PCS or MCS scores over the 16-week study period. The intervention also did not lead to significant changes in self-care markers compared to control.

Two of the themes that emerged from qualitative interviews were: the convenience of delivering the intervention during hemodialysis and the novel content of the intervention. Participants were engaged with sessions because they had few competing demands during dialysis. Occasionally, however, they were too tired to participate. Participants also liked the behavioral content of the intervention. They felt that it focused on topics not traditionally covered by social workers, including how dialysis affects their personal lives. They also liked that the homework reinforced behavioral techniques.

Recommendations for adapting the intervention emerged from qualitative interviews. One social worker felt there were too many self-care recommendations delivered over the 8 sessions. She suggested focusing on a few topics. Physicians noted the difficulty in retaining social workers to deliver the intervention and recommended that other health care providers be trained to deliver the intervention. They felt that being engaged with patients and being dedicated to delivering the intervention without other responsibilities were the only requirements necessary.

Two prior randomized controlled trials (RCT) of CB interventions in patients on dialysis with depression successfully improved depressive symptoms, QOL, and self-care (intradialytic weight gain) (10, 11). Both interventions were delivered by a psychologist. One delivered individual CBT during the HD session, while the other delivered group CBT at a time when participants were off dialysis. To our knowledge there is only one prior study of a behavioral intervention that targeted all dialysis patients regardless of the presence of depression and that targeted improvements in QOL and self-care [25]. In a RCT of 56 patients on HD, Sharp et al. studied the effect of a 4 weekly hour-long group CB sessions, delivered by a supervised trainee psychologist in the outpatient setting, on improving fluid-restriction in dialysis patients that had an average daily IDWG of > 2.5kg. The authors found improved adherence to fluid restriction and in some cognition that may mediate this improvement but no improvement in psychosocial functioning.

There are differences between our intervention and the aforementioned study. Unlike the prior study, we chose to do the intervention individually and chair-side during dialysis. Although this was done to optimize adherence to sessions and prevent drop-out, it may have prevented beneficial group interactions. Additionally, instead of using a psychologist, we trained a dialysis social work to deliver the intervention. A similar technique has been used successfully in other chronic illnesses. In a randomized trial of 222 patients with poorly controlled diabetes with minimal complications, a group-based behavioral-education, delivered by nurse and dietician educators trained to deliver CB strategies, significantly improved HbA1c compared to control arms without these strategies [16].

We suggest two major changes for future studies of our intervention. The first would be to choose a nurse, nutritionist, or dialysis technician that has been certified in ESKD education, without other conflicting responsibilities, and that has been trained in delivering CB strategies, to deliver the intervention. This would allow a larger group of people to deliver the intervention. The second change would be to narrow the scope of the sessions to focus on only a few aspects of self-care.

The strength of this study lies in its simplicity and its potential to be immediately implemented in US HD units. It is brief and performed during the HD session by dialysis social workers. Additionally, the slides, worksheets and handouts are readily available.

The study had several limitations. There was turnover in the social workers performing the intervention and this delayed the time from randomization to the first intervention session, leading to unequal drop-out among groups and limiting our results. As a pilot study, we analyzed only data from subjects that completed at least one intervention or control session. This likely introduced bias but enabled us to get more data about the effectiveness of the intervention. Additionally, we were not able to record intervention sessions due to constraints placed by the dialysis units and dialysis clinical social workers only received 6-hours of training in CB-strategies potentially limiting the behavioral interventions performed with participants. Finally, the study was underpowered. The original power analysis determined that 40 participants were needed to detect clinically relevant changes in HrQOL (8-point change in PCS), and, due to participant and social worker drop-out, only 34 participants were analyzed. There is, however, important data regarding the feasibility of the intervention that can be used for future studies.

## Conclusions

In summary, we were able to test the feasibility of a simple CB-based behavioral-education to improve quality of life and self-care in patients on HD. There were several positive aspects of the intervention including its chair-side delivery and the inclusion of content related to the psychological challenges for patients on dialysis. Through our experience, we are now able to modify our intervention for future implementation.

## Supporting information

S1 Checklist Coplon consort. (PDF)

S1 File Protocol clean. (DOCX)
