# Supplementary material for: Improving quality of life and self-care for patients on hemodialysis using cognitive behavioral strategies: A randomized controlled pilot trial
Source: PLoS One. 2023 May 4;18(5):e0285156. doi: 10.1371/journal.pone.0285156 (PMC10159130; doi:10.1371/journal.pone.0285156)
Supplement: S1 File — (DOCX) [file pone.0285156.s002.docx]

Short Title: Improving Outcomes for Hemodialysis Patients

**Full Title: Improving Outcomes for Hemodialysis Patients by Addressing Poor Health Related Quality of Life with Cognitive Behavioral Strategies**

**Institution**: Columbia University Medical Center

Study Site: Rogosin East Hemodialysis Center

Rogosin West Hemodialysis Center

Columbia University Dialysis Center

**Investigators:** Shayan Shirazian, MD (PI)

**Arlene Smaldone, PhD**

Maya Rao, MD

**Funding**: Satellite’s Norman S. Coplon Applied Pragmatic Clinical

Research Grant

**OBJECTIVE #1: Conduct a randomized controlled trial in 40 hemodialysis patients with below average health related quality of life (HrQOL) scores to determine the impact of a multi-faceted self-management intervention on HrQOL, depressive symptoms, self-management, and hospitalization rates.**

**OBJECTIVE #2: To refine our intervention, using end of study qualitative data, and to develop a translatable toolbox that helps other hemodialysis units implement our intervention.**

**BACKGROUND AND RATIONALE:**

Hemodialysis (HD) patients have worse health related quality of life (HrQOL) than patients with any other chronic illness.^1^ In this population, poor quality of life and depressive symptoms (a major component of HrQOL) are associated with medication non-compliance, dietary indiscretion, interdialytic weight gain, and missed dialysis.^2^ These associations likely explain the link between low HrQOL scores and adverse medical outcomes.^3, 4^ **Even small decrements in HrQOL score are associated with an increased risk of hospitalizations and mortality.**^5^ Moreover, depressive symptoms occur in one-third of HD patients and are themselves associated with increased hospitalizations and with a 1.5 times higher mortality risk.^2^

To address poor HrQOL in HD patients, the Centers for Medicare Services (CMS) mandated its screening on a regular basis in all HD patients.^6^ However, CMS has not mandated how to present HrQOL results to key HD providers, or how to treat those with poor HrQOL. Nor are there widely applied methods of doing so. Although prior interventions in HD patients have improved quality of life and self-management, these interventions were limited by poor patient and physician adoption, a lack of reproducible methods, selective inclusion and exclusion criteria and a lack of translatability.^7-10^ **To date there are no widely adopted interventions to improve quality of life in this population.^6,11^** Thus, it is not surprising that, HrQOL scores remain unchanged in 8 years since the CMS mandate.^11^ In sum, HrQOL survey results have not been applied in a manner that makes a difference for patients.

Cognitive behavioral therapy (CBT) is a structured psychotherapy intervention designed to address and treat dysfunctional cognitions, negative emotions and maladaptive behaviors. In patients with chronic illness, CBT has been adapted to successfully improve adherence to self-management behaviors.^12-14^ In patients with ESRD, several studies have used cognitive behavioral strategies to improve self-management, quality of life, and depressive symptoms.^13,14^ These studies however were limited by high drop-out rates and a lack of translatability. To date, cognitive behavioral (CB)-strategies are not routinely used in the care of ESRD patients.

**The investigators have developed a simple, translatable 3-step intervention to improve poor HrQOL in hemodialysis patients**. The first step is a one-page dashboard that presents actionable HrQOL data to key stakeholders (social workers, nutritionists, primary nurses, nurse-practioners, physicians, patients and family members) during monthly case conference. The second step is a treatment approach that combines self-management education with CB strategies through 8-12 simple sessions delivered chair-side over 12 weeks. These sessions will be delivered by unit social workers and are designed to be highly translatable to other US hemodialysis units. The final step is monthly re-evaluation at clinical case conference where patient progress will be assessed and the dashboard and behavioral-education sessions refined. Each step of the intervention was designed to be highly translatable to current hemodialysis care. The dashboard utilizes data that is currently checked, the treatment sessions are delivered by social workers that are already employed at hemodialysis centers and re-evaluation occurs during monthly case conference sessions that are already being held.

**The investigators hypothesize that implementing their multi-faceted intervention will improve HrQOL, depressive symptoms and self-management, and will be immediately translatable to other US hemodialysis units.** Therefore, the investigators propose to conduct a 16-week randomized controlled trial in 40 subjects on hemodialysis with poor HrQOL to test the impact of the intervention on these outcomes. The primary outcome, Kidney Disease Quality of Life Short Form 36 (KDQOL-36™) survey scores, will be measured at 0, 8, and 16 weeks. Through end-of-study focus groups, we will refine our intervention and help translate our intervention into practice. Additionally, we will develop a translatable toolbox that will give step-by-step instructions on how to implement our intervention at other US hemodialysis units. Finally, we will explore the effect of our intervention on hospitalizations.

**STUDY DESIGN**

The study will be designed as a 16-week randomized controlled trial in which 40 subjects on hemodialysis with below average HrQOL will be assigned to our multifaceted intervention vs. usual care with dialysis education (control group).

**NUMBER OF SUBJECTS: 50 (**40 are anticipated to complete the intervention)

**STUDY DURATION**

This study will take place over a 2-year period. See timeline below.

| **ACTIVITIES** | **Months 0-3** | **Months 3-6** | **Months 6-15** | **Months 15-18** | **Months 18-21** | **Months 21-24** |
| --- | --- | --- | --- | --- | --- | --- |
| Recruit subjects | X |  |  |  |  |  |
| In-service dialysis staff and train social workers | X | X |  |  |  |  |
| Conduct trial |  | X | X |  |  |  |
| Qualitative data gathering |  |  |  | X |  |  |
| Analyze data and prepare toolbox |  |  |  | X | X |  |
| Manuscript preparation |  |  |  |  |  | X |

**PRESCREENING, PATIENT ELIGIBILITY AND ENROLLMENT**

Patients on hemodialysis at the study dialysis centers will be pre-screened for eligibility (Form D) from the outpatient medical record at these sites. Prescreening will take place one hemodialysis shift at a time. Initial eligibility criteria will be reviewed and only limited protected health information (name, medical record number and address) and bolded eligibility criteria from the list below will be collected for these pre-screened patients. This pre-screening information will be entered into REDCap (see data management section for further details).

Eligibility criteria include: 1) **On hemodialysis for at least 3 months**; 2) **Most recent 36-question kidney disease quality of life (KDQOL-36) physical component summary (PCS) or mental component summary (MCS) score below 50**; 3) **KDQOL-36 burden of disease score ≤ 80**; 4) Expected survival ≥ 6 months; 5) **English-speaking**. Patients will be excluded if they have: 1) Bipolar or Psychotic disorder 2) Moderate or severe cognitive impairment as determined by the hemodialysis staff or documented in the electronic medical record (EMR) 3) Severe vision or hearing impairment 4) Drug or alcohol dependence; 5) Active suicidal ideation or a history of suicide attempt (determined based on screening patient health questionnaire-9 and EMR) 5) Current participation in a behavioral or education treatment program.

For those patients that meet initial, limited eligibility criteria, the treating nephrologist will be contacted to determine the suitability of the patient for study recruitment. If the patient is deemed a good study candidate, a letter explaining the study and signed by the treating nephrologist will be sent to the patient. The letter will give the patient an option to opt-in to study contact (Appendix 1). Additionally, members of the patient’s treatment team (nephrologist, dialysis nurse, nutritionist or social worker) will approach the patient, after the letter has been sent, to determine study interest. These members of the health care team may distribute a tri-fold (Appendix 2) that further explains the study.

In addition to the recruitment methods described above, study contact will be made via a research flyer posted in the waiting room of outpatient hemodialysis centers, (Appendix 3), and directly by hemodialysis physicians for patients thought to be good study candidates. Any physician contact will be documented by the study team.

An alphabetical list of patients that initiate study contact will be generated.

Study investigators will approach these patients in top down order, one hemodialysis shift at a time. Study investigators will use a written recruitment script (Appendix 4). For patients that refuse to participate, reasons for study refusal will be recorded (Appendix 4).

**STUDY PROCEDURES**

**Electronic Informed Consent (eConsent)**

All eligible patients will go through formal processes of informed consent prior to any study procedures. All eligible patients will be encouraged to complete their consent forms electronically through the RedCap platform. After initial approach by the study staff, potential participants will be given an iPad to view the study consent while they are receiving hemodialysis therapy. They can then read and electronically sign the consent form with an e-signature (tracing their signature with their finger on the iPad) or a copy of the consent will be emailed to them so they can sign remotely (either with their finger or a mouse). For all electronic consents, there is a section at the top of the consent with names, numbers and email addresses to contact if the eligible patient has additional questions about the study or consent process. There is no supplemental media, links or additional resources on the eConsent that explain the study or consent procedures. All eligible patients will also be given the option to sign a hard copy of the consent either at the hemodialysis unit or at home.

Once signed, one copy of the eConsent will be either be emailed to the participant or given as a paper copy. Additionally, a paper copy of all consent forms will be kept in a locked cabinet in the office of the principal investigator and will only be accessible to him.

**Screening / Baseline Dataset**

After informed consent is obtained, demographic, socioeconomic and clinical variables will be collected from all enrolled subjects from the outpatient EMR going back 3 months prior to study enrollment. Additionally, all subjects will complete a demographic, clinical and socioeconomic survey to supplement data not available in the EMR (Appendix 5). Subjects will then complete week 0 surveys including the KDQOL-36 and the patient health questionnaire-9 (PHQ-9) (Appendix 6 and 7). They will be encouraged to use an iPad to complete their surveys directly into RedCap without additional input from study investigators. If this is not feasible for the patient, they will be given paper surveys to complete (see data management section for further details).

All surveys will be completed using a RedCap assigned subject ID number and will not contain any PHI. **Those subjects that do not meet all eligibility criteria (as described above) after screening procedures will be screen failed.**

**Randomization**

Randomization procedures will be performed using a computer-generated 1:1 simple randomization scheme using RedCap. Randomization will ensure approximately equal groups (intervention vs. usual care). All data will be analyzed in a blinded fashion.

Study subjects will not explicitly be made aware of what study group they have been randomized to, however providers will be made aware of study group assignment when they are solicited for input on study curriculum during monthly case conference. Study investigators will also be aware of study group assignment.

**Study Schedule**

All study subjects will follow the same schedule after arrival to the hemodialysis unit: surveys (weeks 0, 8, 16), and intervention or usual care sessions (8-12 times over 12 weeks). Subjects will be encouraged to complete surveys entirely on their own, directly into the RedCap system, without input from study investigators. Each study day will last approximately 30-40 minutes. A total of 8-12 study sessions will be delivered over the first 12 weeks of the 16-week study period. At week 16 surveys will be performed; however, no intervention or usual care sessions will be conducted. In order to reinforce subject retention, study staff will call and remind subjects of study appointments the day prior. Subjects will be compensated $60 for study participation to cover their travel to monthly case conference. If they fail to complete the study, they will receive an appropriate pro-rated amount.

Intervention


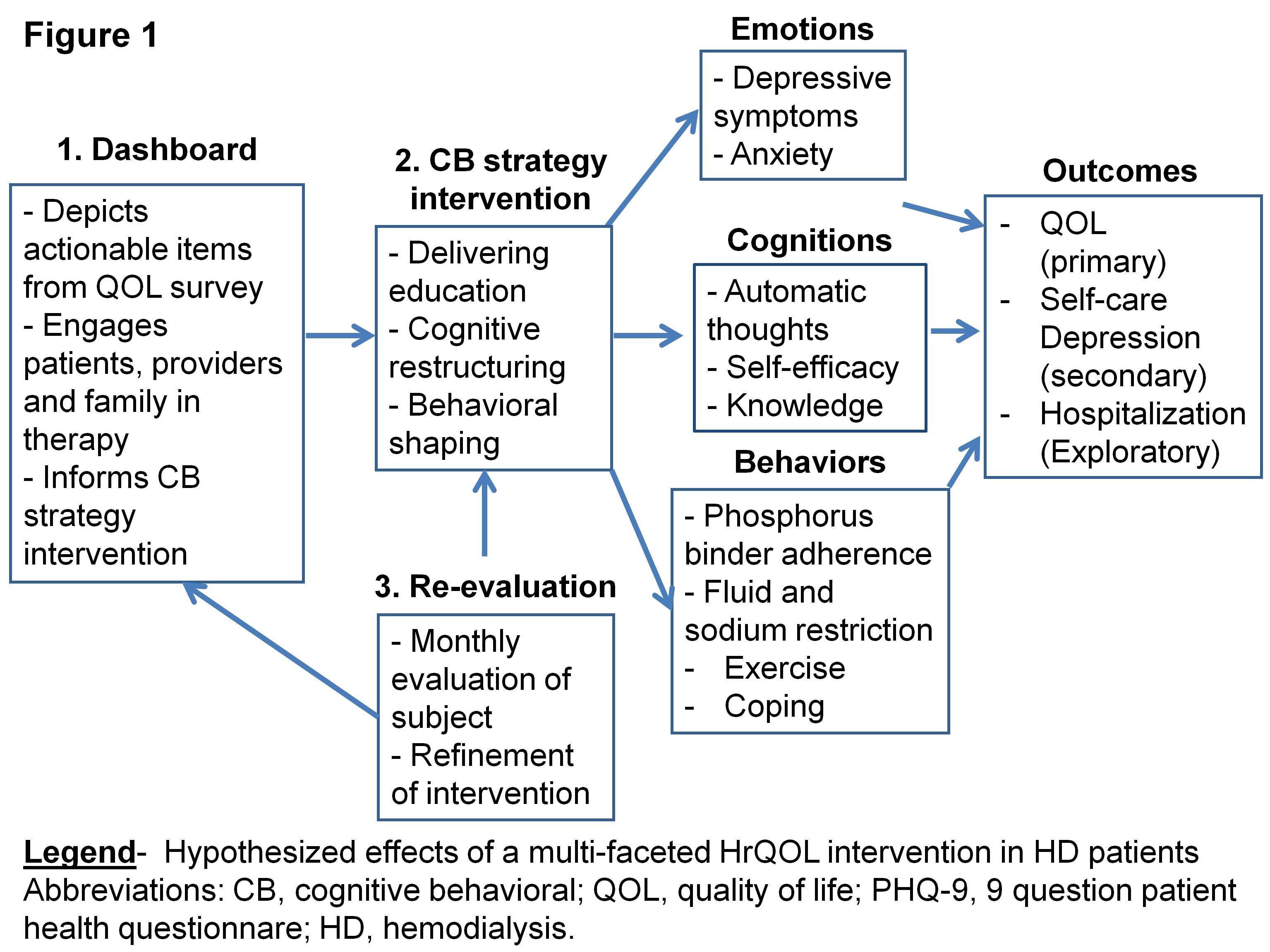
 This study tests a 3-step intervention to treat poor HrQOL in patients on hemodialysis (Figure 1). The first step is a simple, one-page dashboard that displays HrQOL results to patients, family members and the HD treatment team during monthly case conference, the second step is an approach to improving self-management with education combined with CB strategies, and the third step is monthly re-evaluation of patient progress at case conference with all the key stakeholders present. Each step is described in further detail below

*1) Dashboard Design:*

The dashboard has three sections (Appendix 8). The first was designed to quickly highlight actionable items from the HrQOL survey for the treatment team. These are not raw scores but include actual answers from the KDQOL-36 survey. These actionable items will include answers addressing difficulties with coping from the burden of disease sub-section, answers addressing problems and symptoms related to dialysis and answers addressing difficulties with diet. The second section of the dashboard includes recent results that gauge the subject’s self-management. This will include monthly average pre- hemodialysis potassium and phosphorus levels, and monthly average inter-dialytic weight gain. The final section will include education and treatment strategies that will be delivered as part of the subject’s behavioral education intervention (described further below). The dashboard will be presented to the treatment team, subjects and family members during monthly case conference. During this conference, group decisions will be made regarding the CB-intervention educational content and approach. During each subsequent monthly case conference over the 16-week study period, the dashboard will be discussed and the study intervention will be revised as needed. The CB-intervention will begin within 1 week of the first case conference.

*2) Design of CB Strategy Intervention:*

We designed our treatment approach to be similar to the successful structured behavioral intervention designed by Dr. Weinger (a study consultant) in subjects with diabetes.^12^ The intervention will consist of eight to twelve, 20-30 minute, face-to-face, one-on-one sessions preferably delivered chair-side in a private room during hemodialysis over 12 weeks. If a private room is not feasible, other options for the location of the intervention include chair-side in the main dialysis room or in a separate dialysis conference room preferably before treatment. Subjects will be encouraged to bring a family member to each session. If this is not logistically feasible, a family member can participate in the intervention via speakerphone. The sessions will be run by a licensed clinical social worker with experience delivering therapy. This social worker is currently employed by the hemodialysis unit and has at least 3 years of therapy training under a licensed clinical social worker. She also currently delivers education for patients that are found to have poor quality of life (see control group section). This social worker will undergo an initial 6-hour training session directed by Dr. Weinger on CB strategies and a follow-up 3-hour reinforcement session. In order to ensure the fidelity of delivered sessions, a random sampling of five CB- sessions will be audiotaped (described for subjects in the initial consent form). Additionally, the principal investigator may attend up to five CB-sessions. If any variability in CB training is noted, additional CB-training sessions will be delivered by Dr. Weinger.

The CB sessions curriculum will introduce education relevant for self-management and behavioral techniques to overcome barriers to self-management, and consists of prepared slides, handouts and worksheets (see Appendix 9). The education was designed based on slides developed and published online by the national kidney foundation and the national kidney disease education program,^15^ based on preliminary focus groups, and based on patient needs assessed during monthly case conference by the treatment team with the aid of our dashboard. The education derived from outside sources is either publicly available free of charge or permission was obtained to include the materials. The curriculum was designed to be understandable by all potential subjects including those with low literacy levels and to be immediately publishable after the study intervention.

Possible education categories include potassium control, phosphorus control, sodium and liquid control, activity, medication adherence, physician follow-up and coping. Patients will likely need education from more than one category. This will be decided during their monthly case conference. This is why the sessions can range in number from 8-12. All education sessions will incorporate CB strategies to overcome barriers to self-management (see below). All patients that have difficulty coping or depressive symptoms will receive 1-2 CB strategy based sessions that address coping with the physical demands of self-management, dialysis treatment and the emotional demands of having multiple comorbid illness (example slide shown in Appendix 10). These sessions will include discussions of perceptions surrounding ESRD, including distress related to poor quality of life. Common barriers to ESRD self-management will be addressed and potential CB strategies to overcome these barriers will be presented.

All sessions will end with homework. This content will be individualized for the patient. Examples of homework assignments include identifying risk factors for non-adherence to self-management recommendations (for potassium, phosphorus, salt and liquid restriction, or physical activity as appropriate for each individual patient), identifying barriers to self-management, problem solving and goal setting exercises (see below), and the creation of ongoing self-management logs including diet, exercise, and medication logs.

All sessions will incorporate behavior-based activities designed to deliver education about and improve upon medication adherence, diet, exercise, and coping. These sessions will include discussions and activities aided by worksheets and handouts (Appendix 9). These activities, based around CB strategies such as cognitive restructuring and behavior shaping, will include: 1) review of self-management logs, 2) goal setting, 3) creation of treatment plan, 4) problem-solving techniques, 5) reinforcing techniques, and 6) coping with ESRD. These activities have successfully improved chronic illness.^16^ See additional description of these activities below:

*Thematic Element: Goal Setting and Shared ESRD Treatment Plan.*

For each subject, areas that are not at goal from monthly case conference and from self-management logs will be identified and designated as problem areas. The social worker will then help subjects set self-management goals and a strategy for achieving these goals. This “ESRD treatment plan” will be individualized and will utilize CB strategies for achieving targets. The social worker will work individually with study subjects to revise ESRD treatment plans, making them more feasible. Finally, the social worker will stress making mutual decisions about this plan with their care providers and will teach subjects how to revise their treatment plans according to changing goals and life circumstances.

*Thematic Element: Problem Solving.*

For each subject, barriers that hinder ESRD treatment will be identified. The social worker will teach CB-based problem-solving techniques to overcome these barriers and will suggest CB-based solutions to overcome these barriers.

*Thematic Element: Reinforcing Techniques.*

For all sessions, personal barriers to achieving ESRD goals will be discussed and behavioral techniques used to overcome these barriers will be reinforced. ESRD treatment goals will also be continually analyzed and revised to make them more feasible for the patient.

*Thematic Element: Coping.*

Subjects that express negative reactions to their ESRD status and its treatment will be taught reframing techniques to help develop healthier reactions to illness.

*Reinforcement sessions:*

After the initial educational or coping content is presented, patients will intermittently receive brief (5-10 minute) reinforcement sessions on key educational content. These reinforcement sessions will vary in number based on subject progress. Those subjects that do not appear to be progressing with treatment will receive the maximum number of study sessions.

3. *Monthly Progress Evaluation:*

Monthly re-evaluation of intervention subjects will occur at case conference with all the key stakeholders present. The subject’s dashboard will be re-evaluated, including the trend of HrQOL and PHQ-9 scores and the trend in self-management indicators. This information and input from the patient and family member will help the treatment team decide on the content and number of further CB-education sessions.

**Control Group:**

The control group will continue to receive the usual care for low HrQOL scores as dictated by unit policy with additional self-management education delivered by a study investigators. Usual care includes social work review of HrQOL scores, and use of a template to report scores back to patients. Social workers review these results with the patient and give their own suggestions on how to improve HrQOL. These suggestions include increasing activity and adjusting diet if PCS is low, seeking treatment for depression or anxiety or being more social if MCS is low and staying positive if their burden of kidney scores are low. The control group subjects will also have access to all the educational material from the study intervention. Distribution of this material will be left to the discretion of the study investigator assigned to the control group. This contact with the study investigator will be limited to 8-12, twenty to thirty minute sessions over the study period. Additionally, social workers that deliver the study intervention will not be permitted to deliver control group sessions in order to avoid contamination between groups. The control group will also continue to receive monthly case conference with all the key stakeholders present, as per unit protocol

**OUTCOMES**

The outcomes will be measured at weeks 0, 8, and 16

**Primary Outcome:**

The primary outcome will be the change in HrQOL scores over the 16-week study period. HrQOL will be measured using the Kidney Disease Quality of Life Short Form 36 (KDQOL-36™).^17^ KDQOL-36 is a self-report questionnaire that has previously been validated in multiple chronic illnesses including patients with chronic kidney disease of multiple different ethnicities.^17^ The survey contains 5 subscales: the SF-12 Physical Component Summary (PCS) subscale (Questions 1-12), SF-12 Mental Component Summary (MCS) subscale (Questions 1-12), Burden of Kidney Disease subscales (Questions 13-16), Symptoms and Problems subscale (Questions 17-28), Effects of Kidney Disease on Daily Life subscale (Questions 29-36). The survey is a public document and available free to use without permission.

***Secondary Outcomes:***

*1.* Depressive Symptoms will be measured by the Nine-Question Patient Health Questionnaire (PHQ-9).^18^ This self-report survey is validated in ESRD, and is routinely administered in our hemodialysis unit.^18,19^ Permission is not required to use the PHQ-9 survey. Question 9 of the survey is a question related to suicidal thoughts. Question 9 will be reviewed by a study investigator prior to the patient leaving the hemodialysis unit, and if any patient reports a 1 or higher, a suicide assessment will be performed by study staff. Any subject with a plan to harm him or herself will be sent to the psychiatric emergency room, other patients will be referred for psychiatric services and given a number for a suicide hotline.

Self-management Indicators:

1. Potassium and phosphorus control will be measured by the mean of all pre-HD potassium and phosphorus values obtained monthly in the 3 months prior to the intervention and obtained monthly during the last 3 months of the study intervention.
2. Interdialytic weight gain will be measured as the average weight gain between hemodialysis sessions in the 3 months prior to the study intervention and during the last 3 months of the study intervention.
3. Skipped hemodialysis sessions will be measured in the 3 months prior to the study intervention and during the last 3 months of the study intervention. A session will be considered as skipped if the absence was not planned or caused by a hospitalization or illness

In order to gauge the acceptability of our intervention, a flowchart of patients approached and those consented will be recorded. We will elicit reasons for study refusal from those that decline to participate. Additionally, a detailed log of homework assignments completed will be kept.

**Feasibility Outcomes:**

A flowchart of patients approached and those consented will be recorded. We will elicit reasons for study refusal from those that decline to participate. Additionally, a detailed log of completed homework assignments will be kept.

**Exploratory Outcome:**

Hospitalizations including the discharge diagnosis, days in the hospital and outcome will be determined by detailed clinical chart reviews performed by a trained nephrologist with over 5 years of clinical experience. This investigator will be blinded to study group assignment. The type of hospitalization will be adjudicated as heart failure, access failure, access infection, other infection, coronary artery disease related, hyperkalemia, missed dialysis, or other.

**SURVEY TIME:**

In total, there will be 73 survey questions for subjects at week 0 which will take approximately 30 minutes to complete. At weeks 8 and 16 subjects will take a total of 45 survey questions which will take approximately 20 minutes to complete.

**SAMPLE SIZE:**

To examine the power to detect clinically relevant intervention-related changes in HrQOL outcomes, we simulated normally distributed pre- and 16-week post intervention HrQOL scores with no changes in mean HrQOL in the control group and an absolute increase of at least 8 points in the MCS or PCS (from pre to post) in the intervention, a within-group standard deviation (SD) of 10.0 and positive correlation between HrQOL measurements from the same person such that Pearson’s rho=0.70. Our hypothesized effect size was based on studies showing that a change of 8 points or more in PCS has been associated with a clinically significant decrease in hospitalizations and mortality.^1, 2^ Behavioral interventions have shown similar improvements in HrQOL over 3-4 months.^3, 4^ **With n=20 per treatment arm, we have 85% power at the 0.025 level of significance to detect clinically relevant changes in HrQOL**.

**DROP-OUT**

High drop-out rates for studies of self-management and psychiatric interventions have been reported.^5^ We will recruit subjects until 40 complete the intervention.

**ANALYSIS PLAN:**

Data will be analyzed on an intent-to-treat basis. All data will be analyzed in a blinded fashion. For each HrQOL outcome, we will summarize the average changes from pre to post for each treatment arm via means (±SD) and 95% confidence intervals and estimate the effect of our intervention by estimating a linear model for each post-treatment score, adjusting for pre-treatment score and treatment arm (i.e. an ANCOVA model). The point estimate, confidence interval, and statistical significance of the treatment arm parameter will be retained from the model and evaluated for statistical significance. A significance level of 0.025 will ensure that the family-wise error rate will be ≤5%, thus two-sided p-values less than 0.025 will be considered statistically significant. Each model’s residuals and diagnostic plots will be checked to make sure that model assumptions are reasonable. If model assumptions are not fully met, non-parametric approaches will be employed in the primary analysis and the consistency of results evaluated. In secondary analyses, depressive symptoms and self-management behavior endpoints will be summarized for each treatment arm via means (±SD) and 95% confidence intervals. All statistical analysis will be conducted using SAS/STAT software (Version 9.1, Cary, NC, USA).

**Objective #2: To refine our intervention, using end of study qualitative data, and to develop a translatable toolbox that helps other hemodialysis units implement our intervention.**

We will use qualitative means, including focus groups and semi-structured interviews to refine our intervention and develop a translatable toolbox.

**DESIGN:**

End of study focus groups of subjects, and family members will be structured to assess the appropriateness, barriers, facilitators and the ability for translation of the intervention. These focus groups will be structured according to a RE-AIM framework (reach, effectiveness, adoption, implementation, maintenance) in order to help translate the intervention into clinical practice.^20^ A preliminary interview guide has been provided in Appendix 11. All intervention subjects are eligible for participation in the focus groups and will be asked to participate with the family member that participated in the intervention with them. Focus groups will consist of 6-8 participants and will be performed within 6 weeks following the study intervention. We anticipate there will be 4-6 focus groups. Additionally, health care providers with exposure to the study intervention will be asked to participate in semi-structured interviews. These semi-structured interviews follow the same framework and interview guide as the focus groups. We anticipate approximately 4-5 semi-structured interviews. Both focus groups and semi-structured interviews will be led by a moderator. Family members and health care providers will be given a one-page information sheet describing the focus group/interview prior to their participation, in lieu of formal consent (Appendix 12).

At the end of the study, investigators will meet to discuss the study findings and themes stemming from the qualitative data analysis. Revisions to the study curriculum will be made in response to these data with the goal of making the intervention as simple and generalizable as possible to the largest number of hemodialysis patients. The study intervention content will then be made into a form that is immediately externally publishable for other dialysis centers. This content will include PowerPoint slides for providers, handouts for patients, and a video training in CB strategies led by Dr. Katie Weinger an expert in CB training. The end of study content in its publishable form will be termed the CB-HD toolbox.

**Statistical Analysis of qualitative data**

Qualitative data will be interpreted through thematic analysis. Audio files will be transcribed by a transcription service. Transcripts will then be uploaded into NVivo, a qualitative software program for coding and theme generation. After coding is complete, the study team will meet to read through the transcripts again, and group the codes into themes and an overarching framework. Credibility (validity) will be supported through triangulation of data sources (transcripts, field notes, and observation) and the use of multiple investigators from different disciplines. Dependability (reliability) will be established through an audit trail of reflexive and operational memos.

**Potential Benefits**

The study program has the potential to improve mood, self-management and medical outcomes. Additionally, subjects that request results of their testing will receive a health assessment including clinical and biomedical data, which may identify potentially problematic areas for the subject. Subjects that request results of the psychosocial measures will be provided a brief summary and will be given the opportunity to review their results with the study investigators.

Finally all subjects enrolled in the pilot trial will be reimbursed $60 for their study participation. If the subject does not complete the study, they will receive a pro-rated amount to cover costs related to their travel. Additionally, all subjects that participate in focus groups or interviews will be reimbursed $20 for their time.

**Potential Risks**

The study program or the psychosocial surveys that are administered may trigger emotional distress especially when sensitive topics are addressed (e.g. suicidality, substance abuse). Our study staff including social workers, the coordinator and research assistant will be trained to address any important issues that arise and if needed subjects will be given the opportunity to discuss their concerns with a licensed social worker. Additionally, the study program will be performed by a social worker that has been trained to deal with situations that may put subjects in immediate danger including (suicidality). She will make appropriate referrals for these subjects including for emergency psychiatric services if needed. If for some reason she is not available and subjects are deemed to be in immediate danger by the study staff (suicidality, or homicidality), they will be referred for emergency psychiatric services. Study staff will participate in training including didactic materials, role-play and supervised practice, to ensure subject concerns and dangerous situations can be addressed quickly and appropriately.

Additionally, adverse events will be reported by the study team on an ongoing basis, and any unanticipated problem or serious adverse event will be reported to the Columbia IRB.

**Confidentiality and Storage of Data**

A customized, secure, web-based data management system (REDCap) has been designed to run at Columbia University Medical Center. REDCap is designed to be HIPPA compliant and all web-based information transfer is encrypted. All study data including protected health information (PHI) will be stored in the REDCap database. Only the principal investigator, research coordinator and social worker will have access to this information in RedCap.

PHI in RedCap will be linked to subject ID numbers. Whenever study data is downloaded from RedCap, PHI will be hidden and only the linked subject ID numbers will be used. Subjects will have the opportunity to complete surveys using their linked ID numbers directly into REDCap during their study visits with an iPad, or, if this is not possible, hard copy surveys will be completed by subjects using their linked ID number and this data will be entered into REDCap by a trained investigator. For all entered data, fields will be reviewed for out of range values. If missing fields or illogical values are encountered, the study coordinator will review the original source documentation with the data abstractor and make corrections as appropriate. The de-identified study data will be directly exportable to SAS/STATA for statistical analysis. Any paper/hard copy data and study materials with subject ID numbers will be stored securely in a locked file cabinet in the Columbia Division of Nephrology offices. One paper copy of the subjects consent form, with the subject’s name, will be stored securely in a locked file cabinet in the Columbia Division of Nephrology offices. One copy of this consent will either be emailed or given as a paper copy to the subject.

All Columbia RedCap data is backed up daily, and backups are stored for 8 weeks. In addition, the principal investigator will keep one copy of all study data on a password protected, encrypted computer in his office. This file will be updated and overwritten by the PI on a weekly basis.

Clinical information will not be released without written permission of the subject, except as necessary for viewing by study investigators and monitoring by IRB, the FDA and the Office of Health Research Protection OHRP. All data will be stored using protective procedures described above and maintained for a minimum of three years after the completion of the study.

**REFERENCES**

1. Mittal SK, Ahern L, Flaster E, Maesaka JK, Fishbane S. Self-assessed physical and mental function of haemodialysis patients. Nephrology, dialysis, transplantation : official publication of the European Dialysis and Transplant Association - European Renal Association. 2001;16(7):1387-94.

2. Shirazian S, Grant CD, Aina O, Mattana J, Khorassani F, and Ricardo AC. Depression in Chronic Kidney Disease and End-Stage Renal Disease: Similarities and Differences in Diagnosis, Epidemiology, and Management. Kidney Int Rep Jan;2(1):94-107

3. Flythe JE, Kimmel SE, Brunelli SM. Rapid fluid removal during dialysis is associated with cardiovascular morbidity and mortality. Kidney Int. 2011;79(2):250-7. PMCID: PMC3091945.

4. Unruh ML, Evans IV, Fink NE, Powe NR, Meyer KB. Skipped treatments, markers of nutritional nonadherence, and survival among incident hemodialysis patients. Am J Kidney Dis. 2005;46(6):1107-16.

5. Lowrie EG, Curtin RB, LePain N, Schatell D. Medical outcomes study short form-36: a consistent and powerful predictor of morbidity and mortality in dialysis patients. Am J Kidney Dis. 2003;41(6):1286-92.

6. Finkelstein FO, Wuerth D, Finkelstein SH. Health related quality of life and the CKD patient: challenges for the nephrology community. Kidney Int. 2009;76(9):946-52.

7. Mason J, Khunti K, Stone M, Farooqi A, Carr S. Educational interventions in kidney disease care: a systematic review of randomized trials. Am J Kidney Dis. 2008;51(6):933-51.

8. Weisbord SD, Mor MK, Green JA, Sevick MA, Shields AM, Zhao X, et al. Comparison of symptom management strategies for pain, erectile dysfunction, and depression in patients receiving chronic hemodialysis: a cluster randomized effectiveness trial. Clinical journal of the American Society of Nephrology : CJASN. 2013;8(1):90-9. PMCID: PMC3531656.

9. Tsay SL, Lee YC, Lee YC. Effects of an adaptation training programme for patients with end-stage renal disease. Journal of advanced nursing. 2005;50(1):39-46.

10. Mathers TR. Effects of psychosocial education on adaptation in elderly hemodialysis patients. ANNA journal. 1999;26(6):587-9.

11. Chen SS, Al Mawed S, Unruh M. Health-Related Quality of Life in End-Stage Renal Disease Patients: How Often Should We Ask and What Do We Do with the Answer? Blood purification. 2016;41(1-3):218-24.

12. Weinger K, Beverly EA, Lee Y, Sitnokov L, Ganda OP, Caballero AE. The effect of a structured behavioral intervention on poorly controlled diabetes: a randomized controlled trial. Arch Intern Med. 2011;171(22):1990-9. PMCID: 3487475.

13. Sharp J, Wild MR, Gumley AI, Deighan CJ. A cognitive behavioral group approach to enhance adherence to hemodialysis fluid restrictions: a randomized controlled trial. Am J Kid Dis. 2005;45(6):1046-57.

14. Cukor D, Ver Halen N, Asher DR, Coplan JD, Weedon J, Wyka KE, et al. Psychosocial intervention improves depression, quality of life, and fluid adherence in hemodialysis. Journal of the American Society of Nephrology : JASN. 2014;25(1):196-206. PMCID: PMC3871769.

15. National Kidney Foundation - For Patients. (n.d.). Retrieved February 3, 2017, from www.kidney.org/atoz/atozTopic_Dialysis

16. Ellis SE, Speroff T, Dittus RS, Brown A, Pichert JW, Elasy TA. Diabetes patient education: a meta-analysis and meta-regression. Patient Educ Couns. 2004;52(1):97-105.

17. Measuring Dialysis Patients' Health-Related Quality of Life with KDQOL-36TM, 2012. www.kdqol- complete.org/pdfs/kdqol-36.pdf. Accessed February 1st, 2017.

18. Watnick S, Wang PL, Demadura T, Ganzini L. Validation of 2 depression screening tools in dialysis patients. American journal of kidney diseases : the official journal of the National Kidney Foundation. 2005;46(5):919-24.

19. CMS Centers for Clinical Standards and Quality: Summary of Representative Clinical Depression Screening tools. 2015; https://www.cms.gov/Medicare/Quality-Initiatives-Patient-Assessment- Instruments/ESRDQIP/Downloads/Summary-of-Representative-Clinical-Depression-Screening-Tools.pdf. Accessed Feb 1st 2017

20. Glasgow RE, Vogt TM, Boles SM. Evaluating the public health impact of health-promotion interventions: the RE-AIM framework. Am J Public Health. 1999;89(9):1322-27.
